# COVID 19 and Dental Education: Transitioning from a Well-established Synchronous Format and Face to Face Teaching to an Asynchronous Format of Dental Clinical Teaching and Learning

**DOI:** 10.1177/2382120521999667

**Published:** 2021-03-15

**Authors:** Melanie Nasseripour, Jonathan Turner, Susha Rajadurai, Jonathan San Diego, Barry Quinn, Anitha Bartlett, Ana Angelova Volponi

**Affiliations:** 1 Ethics and Dental Education, Centre for Dental Education, Faculty of Dentistry, Oral & Craniofacial Sciences, King’s College London, UK; 2 Clinical Education, Centre for Dental Education, Faculty of Dentistry, Oral & Craniofacial Sciences, King’s College London, UK; 3 Centre for Dental Education, Faculty of Dentistry, Oral & Craniofacial Sciences, King’s College London, UK; 4 Technology & Health Informatics Education, Director of Informatics and Technology Enhanced Learning Hub (iTEL Hub), Centre for Dental Education, Faculty of Dentistry, Oral & Craniofacial Sciences, King’s College London, UK; 5 Simulation and Team-based Clinical Education/Hon Consultant in Restorative Dentistry, Centre for Dental Education, Faculty of Dentistry, Oral & Craniofacial Sciences, King’s College London, UK; 6 Regenerative Dentistry Education, Centre for Dental Education, Faculty of Dentistry, Oral & Craniofacial Sciences, King’s College London, UK

**Keywords:** Dental, clinical, education, Covid-19

## Abstract

The Covid-19 pandemic made it necessary to adopt and establish complete or partial online delivery of our clinical teaching and learning. We developed an alternative approach with a combination of Problem based Learning asynchronous fora and Teacher-facilitated synchronous online discussions. Our aim is to share our educational practice and highlight the requirements and constraints, advantages and challenges of such an approach. It allowed a more student-centred experience, but clinical simulation and face-to-face patient care remain necessary. The Covid-19 pandemic has changed the landscape of dental education for the foreseeable future, with a reduced number of patients in dental clinics. Further study is therefore necessary to understand the lived experience of students and teachers to the adopted online teaching and learning approach.

## The COVID Situation

Prior to the COVID-19 lockdown, in March 2020, dental faculties delivered face-to face teaching sessions for seminars and clinical training. Whilst this was viable in the past, it became necessary during the pandemic for higher education establishments to adopt and establish complete or partial online delivery of teaching and learning.^1^

Dental clinical teaching and learning differs from other health care profession degree programmes in that dental students, very early on in their programme, will be providing irreversible procedures on conscious and communicating patients, under the supervision of clinical teachers, who take on vicarious responsibility for the students’ work. Other health care profession training programmes will initially have their students observe licensed clinicians providing care for patients. Therefore, clinical teaching and learning sessions chair-side with patients is paramount in the dental curriculum and finding suitable alternatives, due to the challenges related to the pandemic, is of the utmost importance in guaranteeing that our graduates are safe beginners.

To this effect the alternative platforms we introduced, to deliver the remote teaching of conservative and minimum invasive dentistry and prosthodontics for Year 2, 3, 4 and 5 dental students, created a virtual environment where clinical discussion and reasoning were maintained, with students feeling safe from making mistakes that can compromise patient safety. When students feel safe to make mistakes, they initiate a deeper learning.^2^

Patient treatments were replaced by online seminars and fora, designed to simulate such clinical cases. We developed an alternative approach very similar to E-learning as applied to surgery teachings in other medical and surgical curricula^3,4^ with clinical sessions replaced with:

- Problem based learning asynchronous fora where students discussed and debated the management of a clinical problem, among peers and teachers online
- Teacher-facilitated synchronous student discussions in debrief seminars using the MS Teams platform.

## Requirements and Constraints

We had to adapt within the following requirements and constraints:

- Work within the existing timetable.
- Core lecture content had to be delivered online.
- Ensure the online delivery adhered to the required learning outcomes.
- Time zone constraints with international students studying from home.
- Technology constraints.
- Training staff in using different online platforms.
- Adaptability to a fluid situation.
- Variety of considerations included group size, start times, incorporating appropriate breaks in activity; as well as type of delivery, synchronous and/or asynchronous. Use of the existing and well-established online virtual campus platform.

To support the newly introduced, online alternative of our teaching and learning activities, we introduced:

- Faculty-wide administration plan.
- Detailed online guidance and training for staff.
- Reflection and self-assessment activities to compensate for missed clinic sessions and to facilitate students’ personal and professional development and higher cognitive functions.
- Evidence Based Approach with review of the Literature.

We have identified the following advantages and challenges to share with dental educators to improve best practice going forward.

## Advantages

### Asynchronous delivery of lectures

- Pre-recorded lectures provided stable, focused and well-captioned online content.
- Content will only need to be reviewed and updated.
- We observed increased efficiency with reduced complexities of timetabling.
- Students continue to have timetabled lecture slots but are able to watch remotely and at their convenience.

### Synchronous delivery of seminars and substitute clinical scenarios

- Small group seminars allowing development of a familiar and comfortable environment to foster participation and engagement.
- MS Teams allows use of a timetable, helping students’ organisation.
- Content delivered in these sessions was short, case based and discursive, avoiding repetition of lecture content, enabling critical discussion.

## Challenges

When technology is allowed to drive e-learning, without any scaffolding or directive, it is often detrimental to student learning.^5^ The substantial adaptation to the online education approach posed direct challenges to our teaching staff, requiring development of new pedagogical approaches as well as substantial IT support.^6^

Students may not have access to ideal learning environments or technology.

## Take Away Message from Our Approach

The approach adopted allowed a more student-centred experience. The absence of a patient meant students were able to make mistakes and learn from them, without patient safety being compromised.

Limitations that we observed in our online approach, mainly related to the absence of actual interaction with a patient, resulting in absence of clinical (hands-on) and communication skill development. Therefore, clinical simulation and face-to-face patient care remain necessary.^7^

We considered it essential to embed a Reflective Practice Review to accompany and monitor our students’ engagement and progress in terms of their clinical knowledge and skills.

Increased time and administrative requirements were observed, as well as the need for technological and pedagogical support.

The new approach enabled us to deliver our existing learning outcomes for clinical sessions except the ones which were specific to direct patient care, such as carrying out clinical procedures and directly communicating with patients in relation to delivery of care.

We also addressed new learning outcomes associated with the online teaching and learning environment, in particular the development of skills for self-directed learning.

The Covid-19 pandemic has changed the landscape of dental education for the foreseeable future, with a reduced number of patients in dental clinics.^1^ We expect a period of limited clinical exposure for students in terms of procedures carried out, indicating an essential need to develop further the dental simulation curriculum.^8^

To explore the insight gained on online teaching in lieu of clinical sessions, we will be conducting a study to understand the lived experience of students and teachers to the adopted online teaching and learning approach.
